# IVF in Costa Rica

**DOI:** 10.5935/1518-0557.20170060

**Published:** 2017

**Authors:** Carlos Valerio, Karen Vargas, Henriette Raventós

**Affiliations:** 1 Escuela de Salud Pública, Universidad de Costa Rica; 2 CIBCM y Escuela de Biología, Universidad de Costa Rica

**Keywords:** Human Rights, right to equality and non-discrimination, right to privacy, the right to liberty, the right to personal integrity, the right to form a family

## Abstract

For 16 years, Costa Rica was the only country in the world that banned IVF, after it had been successfully conducted from 1995 to 2000. It also has been the only country that banned IVF based on the argument that it protects the embryo. After years of conflict, the prohibition has finally been lifted and the first baby girl was born in March 2017. This paper recounts the judicial and legal struggles Costa Rica faced in order to reestablished its IVF program.

## The prohibition and the role of the Inter-American Commission and Court of Human Rights (2001-2012)

The practice of high complexity Artificial Reproductive Technologies (ART) - *In Vitro* Fertilization (IVF) - was prohibited in Costa Rica, after a ruling by the Constitutional Chamber of the Supreme Court in 2001 (Ombudsman Office of Costa Rica. Annual Report. 2014-2015). Low complexity ART, such as artificial insemination (AI) (including heterologous), is an accepted medical practice and had been legal since the seventies. Nonetheless, the technical regulations and protocols of AI were not implemented until 2015, in response to a sextuplet pregnancy in which 5 of the products died during the first 2 months.

After the proscription of IVF in 2001, a group of patients presented their case to the Inter-American Commission on Human Rights. In its merits report, issued in July 2011, the Commission held that, in imposing such a ban, Costa Rica had violated various rights under the American Convention on Human Rights: the right to have one's private and family life respected; the right to start a family; and the right to equality and non-discrimination (United Nations. Universal Declaration of Human Rights, 1948; United Nations. International Covenant on Economic, Social and Cultural Rights, 1966). Despite the Commission's decision, Costa Rica failed to make any significant progress towards compliance, and in July 2011, the Commission submitted the case to the jurisdiction of the Inter-American Court of Human Rights with the purpose of obtaining justice for the victims (Organization of American States. Murillo *et al.*, Artavia case against the State of Costa Rica 2012a).

On December 21, 2012, the Inter-American Court found Costa Rica in breach of the American Convention on Human Rights, ruling that the State's ban on IVF violated the right to privacy, the right to liberty, the right to personal integrity, and the right to form a family, in conjunction with the right to be free from discrimination (Organization of American States. Murillo *et al.*, Artavia case against the State of Costa Rica, 2012a).

First, the Court considered the status of the embryo, finding that an embryo does not fall within the meaning of 'person' and is therefore not protected by the right to life provision under the ACHR (Organization of American States. Additional Protocol to the American Convention on Human Rights, 1988). It defined conception at the time of implantation in the uterus and considered that the rights of the implanted embryo and fetus are progressive during intrauterine development in pregnancy. In reaching this decision, the Court considered the ACHR within the context of international and regional human rights systems, and applied various interpretive approaches. Second, the Court determined that the IVF ban constitutes interference in private life as it restricts autonomous decision-making on treatments concerning sexual and reproductive health. It noted, among other things, that the severe and discriminatory impact of the ban was disproportionate to the legitimate aim sought to be achieved, namely the protection of embryos, as natural pregnancy also involves loss of embryos. In its analysis, the Court referred to the comparative European jurisprudence and practice material set out in INTERIGHTS' brief.

The Court ordered Costa Rica to legalize IVF within six months, to ensure implementation through the regulation of aspects of IVF, to provide free mental health services for the victims in this case as stated in the Costa Rican Constitution, and to implement continued training on reproductive rights for judicial officials throughout the state. The Court's decision was final and binding for each of the 22 countries that accepted its jurisdiction (Organization of American States. Murillo *et al.*, Artavia case against the State of Costa Rica, 2012a).

## The State's response to the order to reinstitute IVF in Costa Rica (2012-16)

After 16 years of proscription, Costa Rica had to bring the country up to international legal and technical standards in a six-month time frame (Ombudsman Office of Costa Rica. Annual Report. 2015-2016).

The first step was to regulate the procedure through legislation in Congress. There was time until July 2013. Nonetheless, at the legislative level, it was impossible to advance neither of the several projects that were presented, discussed and modified in special commissions. None of these projects reached the plenary sessions because of blockage by some legislators with fundamentalist positions, who added so many motions to modify specific articles, that it made it impossible to advance the discussion. The main points in dispute were embryo preservation, gamete donation and access to the procedure by single women and non-heterosexual couples, although the position of some Congressmen was to oppose the order given by the Court and maintain the IVF ban (Ombudsman Office of Costa Rica. Annual Report. 2014-2015).

After three years with no progress, the lawyers of the victims asked for a new audience with the Inter-American Court of Human Rights (IACHR) in 2015 (Organization of American States. Inter-American Court of Human Rights. Murillo *et al*., Artavia case against the State of Costa Rica. Monitoring compliance with the judgment, 2012b). This meeting was held on September 3, 2015, in San Jose, Costa Rica, with representatives of the State, the victims and their lawyers, and the Costa Rican Ombudswoman. Several scientific groups with high legitimacy, such as the Academy of Sciences, the Academy of Medicine and the College of Physicians, had been pressing to comply and some proposed to regulate the practice through a decree, because of the impossibility to regulate through Congress. At the hearing, the Costa Rican State presented a draft of a decree to regulate IVF, which was signed by the President of the Republic a week later (Government of Costa Rica, Executive Decree No. 39210-MP-S, 10 September 2015).

In October 2015, because the decree was contested by the same opposing fundamentalist groups, the Constitutional Court stopped the implementation of the decree until it decided if it was unconstitutional and violated the autonomy of the Social Security Health System. The Attorney General's Office also argued that IVF should be regulated by law. Others, as the Ombudswoman and the Costa Rican National Academy of Science, considered the decree to be enough (Ombudsman Office of Costa Rica. Annual Report. 2015-2016).

In January 2016, the Constitutional Court interpreted, as it did in 2000, that IVF should be regulated by law, since it concerned fundamental rights of the embryo and the right to live. Per resolution Number 1692-2016 of February 2016, "...in the regulation of fundamental rights, the scope of the Executive Branch is highly restricted and a secondary, framework that has been violated by the challenged regulation. While the technique of *in vitro* fertilization does not involve the violation of the right to life with respect to fertilized embryo, as determined by the Commission in the case Artavia Murillo and others *vs.* Costa Rica, the unconstitutionality of the rules of appointment persists, as it is a regulation of fundamental rights beyond the mere establishment of requirements and conditions for their exercise, because the regulations contained affects the content of the right to life and health of women and embryos implanted under the terms set by the Commission, as well as the right to human dignity" (Ombudsman Office of Costa Rica. Annual Report. 2015-2016).

In response, the defense of the victims presented a second lawsuit against the Costa Rican State for not observing the respective resolution to reinstate IVF in Costa Rica (Organization of American States. Inter-American Court of Human Rights. Murillo and others Artavia case against the State of Costa Rica. Monitoring compliance with the judgment, 2012b). In February 26, 2016, the Commission of Human Rights ordered Costa Rica to immediately proceed to make the IVF feasible to 6 couples. On March 1, the Inter-American Court of Human Rights said, that given the obstacles in the legislative process, the Decree should be enough to comply with the order. Without analyzing the fine points of the Decree, the Court noted that the Decree was in accordance with the Resolution of December 2012, guaranteeing access to scientific progress in this matter, and protecting the principle of equality and nondiscrimination. In this regard, the Court reiterated that the measure to regulate should not be an impediment to the exercise of the human rights to private and family life through access to technology. The protection to these rights must have a direct legal effect. Therefore, in the absence of a specific regulation in terms of the Judgment, IVF could be performed and audited with the any technical regulations, protocols, rules and medical standards applicable (United Nations. Observation No. 14 of the Committee on Economic, Social and Cultural Rights of the UN, 2000).

In addition, considering that the Decree was the only possible measure taken by the State to comply with the reparation ordered in the Judgment, and that it is a valid alternative to solve the legal uncertainty, the Executive Decree No. 39210-MP-S remains in force. This, without prejudice to the legislative body if any subsequent regulation should be issued, in compliance with the standards specified in the Sentence.

## The Executive Decree No. 39210-MP-S

The Decree authorizes the implementation of IVF to ensure the reproductive rights of people with infertility. It can be requested by any adult over 18 years of age, with the diagnosis of infertility, either single or in a couple, and as a last therapeutic resource. It can only be performed in medical facilities that meet the requirements defined by the Ministry of Health, as to technological facilities, infrastructure, and an interdisciplinary professional team (Government of Costa Rica. Executive Decree No. 39210-MP-S 10 September 2015).

Both homologous and heterologous forms are recognized, with gamete donation. In accordance with the provisions of article 72 of the Family Code, Law number 5476, the donor does not acquire any rights or obligations inherent to filiation and paternity because of the heterologous IVF technique. For gamete donation, the donor must be 18 or older, not been declared incapable by the courts, and tested to exclude the presence of diseases that could be transmitted to the recipient woman and her offspring. Each approved establishment must have a donor registry, for control of donations. Likewise, the Ministry of Health shall keep a national register of donors, which must contain the information from the records of each establishment and the additional data that the Ministry considers necessary.

The Ministry of Health is responsible for coordinating with the Social Security System and the College of Physicians so that the practice of IVF is carried out according to international standards and human rights. Moreover, the Costa Rican Social Security System (Caja Costarricense del Seguro Social, CCSS, 2016) is obliged to apply IVF and infertility treatment in full respect for human dignity and in conformity with international standards governing the matter.

The decree also includes the obligation to provide complete, clear and understandable information regarding the technique to the subjects, which will then cast their free, voluntary and informed consent prior to application of the technique. The woman undergoing IVF is entitled to receive adequate attention from interdisciplinary approved establishments, to ensure their full physical and psychological state of health. The information contained in the medical record must be guarded under strict confidentiality safeguards as well as the donation process.

As to technical requirements, the decree states that it should follow best international clinical practices. The number of fertilized eggs that are transferred to the uterus of the woman may not exceed two per reproductive cycle. The fertilized eggs that are not transferred in the same cycle will be preserved for future cycles or be donated for adoption. Destruction or donation for experimentation is not allowed.

As for all other regulations that have been discussed in the country, it prohibits the disposal, marketing, testing, fission, genetic alteration, cloning and destruction of fertilized eggs.

## Present state of IVF

The Ministry of Health, the Costa Rican College of Physicians and the CCSS, issued the regulations for health facilities performing assisted reproduction technique. This mandatory regulation establishes the technical and administrative criteria that guide the development of the technique in any public and private institutions seeking to implement IVF.

To comply with the public implementation of IVF in the Executive Order 39210-MP-S, of March 2016, the CCSS approved several draft documents: (a) The protocol for Clinical Care Diagnosis of the Infertile Couple and treatment techniques of Low Complexity in the CCSS Network Health Services; and (b) The protocol of Clinical Care for the diagnosis of the infertile couple and treatment techniques of high complexity in the CCSS health Network Services. Additionally, the CCSS is working on: (a) calculating the total cost on the implementation of IVF and possible funding sources; and (b) the construction of an IVF Laboratory at the Hospital of Women and training of human resources (Caja Costarricense de Seguro Social. Agreement Board of the CCSS, Article 5 of Law No. 8831 meeting held on March 10, 2016; Government of Costa Rica. Executive Decree No. 39616-S, of 11 March 2016; Government of Costa Rica. Executive Decree No. 39210-MP-S, 10 September 2015).

At the private level, two private centers are approved to perform IVF in Costa Rica, and one of them is already implementing IVF. This center announced the birth of the first IVF healthy baby girl on March 8, International Women's Day, of 2017, after 16 years of prohibition (La Nación, 2017).

## Future challenges

The case of Costa Rica is emblematic. For 16 years, Costa Rica was the only country in the world that banned IVF after it had been successfully conducted from 1995 to 2000. It also has been the only country that banned IVF based on the argument that it protects the embryo. After years of conflict, the prohibition has finally been lifted and the first baby girl was born in March 2017 (La Nación, 2017). Nonetheless, there are remaining challenges to be discussed, such as funding in the public sector, possible legal registration issues and IVF medical indications that were not included in our present regulations.

A challenge for a country such as Costa Rica, with a universal healthcare system that has moved through the epidemiological transition to chronic disorders and an older population pyramid with more health-related expenses; an important regional migration pattern which also stresses the finances of the institution; non-payment by employers of their employees' fees, and alleged corruption and embezzlements involving public resources of the CCSS; is how to define resource allocations and priorities in a public health system. How many cycles can be offered? Up to what age? How much increase spending is necessary? How much was already included in the low complexity ART? How many patients will require IVF after low complexity ART? The substantial increase in the cost of health-related expenses globally is also creating a constant pressure on the finances of the CCSS. Healthcare services are also being affected by effectiveness and efficiency issues that are manifested in quality complaints and growing waiting lists. This is a complex political and administrative discussion which is not the purpose of this paper.

As to possible legal problems not contemplated in the Decree, there was no mention on surrogacy and same-sex parenting. Several lawyers consider that what is not specifically prohibited, is legal. Nonetheless, this brings up parenthood registration problems that need to be solved. At present, the woman that has the delivery is the legal mother (even if she is not the donor of the oocyte), unless she gives the baby up for adoption. As these possible cases arise, these problems need to be addressed and solved (Raventós, 2016).

Other accepted indications for IVF in other countries include the preservation of fertility and the prevention of transmission of genetic disorders. The Decree only permits IVF for the treatment of infertility in Costa Rica.

The preservation of fertility in couples or individuals in need of treatment that could compromise their future fertility, because of cancer or other medical conditions, has not been included. In these situations, gametes or embryos can be preserved before fertility compromising treatment, and either fertilized (gametes) or only transferred (embryo) afterwards. Because the regulation in Costa Rica requires a diagnosis of infertility that has not been solved with other treatments, these conditions must be discussed or contested in national or international courts.

Another indication, not included in Costa Rica, is the prevention of the transmission of genetic disorders, a standard practice in many countries. Parents with a dominant or recessive genetic disorder, generally only applicable for Mendelian inheritance disorders with severe health and disability consequences, including early mortality, can choose to have IVF and pre-implantation genetic testing of the resulting embryos, before the transfer of only the embryos without the genetic condition. There are examples were this is extended to include a specific antigen composition so that the baby can cure the genetic condition of an older sib. Selection on other grounds such as sex has been banned, except for the prevention of X linked genetic disorders. Because the regulations for IVF in Costa Rica prohibit embryo manipulation and destruction, and genetic testing would include a biopsy of the embryos to later choose and select the ones without serious medical, in principle these indications would be contrary to the regulations. Nonetheless, it probably will need to be discussed in the future to prevent the birth of children with serious life ending condition for which no treatments are available.
